# Dysfunctional macrophages along the gut-liver axis: a driver of infection in cirrhosis

**DOI:** 10.3389/fimmu.2026.1870116

**Published:** 2026-07-15

**Authors:** Devrim Aslan, Hannelie Korf, Alexander Dumarey, Lena Smets, Ahmed Ghallab, Jan G. Hengstler, Pedro Elias Marques, Schalk van der Merwe

**Affiliations:** 1 Laboratory of Liver Immunology, Leuven Center of Molecular Liver Sciences, Hepatology Research Unit, Department of Chronic Diseases and Metabolism (CHROMETA), KU Leuven, Leuven, Belgium; 2 Department of Gastroenterology and Hepatology, University Hospitals Leuven, Leuven, Belgium; 3 Laboratory for Intravital Imaging and Dynamics of Tumor Progression, VIB Center for Cancer Biology, Leuven, Belgium; 4 Department of Oncology, KU Leuven, Leuven, Belgium; 5 Department of Toxicology, Leibniz Research Centre for Working Environment and Human Factors, Technical University Dortmund, Dortmund, Germany; 6 Forensic Medicine and Toxicology Department, Faculty of Veterinary Medicine, Qena University, Qena, Egypt; 7 Laboratory of Molecular Immunology, Rega Institute, Department of Microbiology, Immunology and Transplantation, KU Leuven, Leuven, Belgium

**Keywords:** bacterial translocation, cirrhosis, gut-liver axis, intestinal barrier, macrophage

## Abstract

Patients with cirrhosis are highly susceptible to infections which could trigger a vicious cycle of events, including hepatic decompensation, organ failure, and increased mortality. These infections often originate from the gastrointestinal tract when bacteria breach the intestinal barrier and disseminate towards the liver. Macrophages are key immune cells along the gut-liver axis, where they play a crucial role in preserving barrier integrity under homeostatic conditions. In cirrhosis, however, macrophage specialization, spatial organization, and antimicrobial functions are disrupted. In this review, we discuss the progress made in understanding the heterogeneity of intestinal and hepatic macrophages, emphasizing their distinct ontogeny and the specialized niches that dictate their function. We describe the roles these populations perform in health, particularly in lining critical cellular barriers within the gut-liver axis, followed by how these processes are compromised in the context of cirrhosis. Finally, we explore how therapeutic approaches might target dysregulated macrophages or restore their barrier-protective functions in advanced liver disease.

## 1 Introduction

Cirrhosis accounts for an annual number of 2 million deaths worldwide, ranking it as the eleventh most common cause of death (1–3). Its prevalence continues to rise, driven mainly by metabolic dysfunction-associated steatotic liver disease (MASLD) and alcohol-related liver disease (ALD, 3). Cirrhosis is defined as a chronic, progressive liver disease characterized by fibrosis and the formation of nodules that disrupt hepatic architecture and impair liver function (4). In advanced stages, acute decompensation (AD) may occur, characterized by complications such as portal hypertension, ascites, hepatic encephalopathy, gastrointestinal bleeding, and infections (4, 5). Notably, infections often lead to sepsis, the development of acute-on-chronic liver failure (ACLF), and multiorgan failure, requiring intensive care unit admission and contributing to a one-year mortality rate of up to 60% (5–7).

Disruption of the gut-liver axis is a major driver of the increased infection rate observed in liver cirrhosis (8–10). The gut-liver axis refers to the bidirectional communication between the gut and the liver through complex metabolic, immune, and endocrine pathways, which is essential for maintaining homeostasis. Under healthy conditions, the intestinal barrier restricts the passage of microbes and toxins into the systemic circulation, thereby protecting the liver from continuous exposure to gut-derived substances (8, 11). Progressive impairment of this barrier in cirrhosis allows bacterial translocation to the systemic circulation through the portal vein. Combined with impaired immune surveillance, this fuels chronic inflammation, contributes to immune cell dysfunction, and exacerbates the risk of infections (9, 12).

Macrophages are key immune cells along the gut-liver axis (8, 13). Intestinal macrophages preserve barrier integrity and prevent bacterial translocation by rapidly clearing invading pathogens through highly efficient phagocytosis and promoting tolerance to commensal microbiota (13, 14). Hepatic macrophages act as the second immune barrier, contributing to inflammatory and antimicrobial responses to gut-derived bacteria reaching the liver via the portal circulation, while also supporting tissue repair and liver homeostasis (15). In cirrhosis, however, these tightly regulated macrophage functions become dysregulated, resulting in impaired pathogen clearance and excessive inflammation (8, 10, 16). The central role of macrophages in cirrhosis is further underscored by the growing interest in macrophage-targeted therapies (17–19). Currently, bacterial infections in patients with cirrhosis are treated with broad-spectrum antibiotics (20).Nevertheless, infection-related mortality remains unacceptably high (20, 21). In hospitalized patients with cirrhosis, infections are associated with a fourfold increase in mortality, with post-infection mortality rates reaching 28% at one month and ˜60% at one year (21, 22). A detailed understanding of macrophage function within the gut-liver axis in cirrhosis may therefore provide a foundation for the development of more effective, targeted pharmacological strategies.

In this review, we summarize the progress made in understanding the heterogeneity of intestinal and hepatic macrophages, focusing on those lining the critical cellular barriers within the gut-liver axis. We highlight recent paradigm shifts in macrophage biology, particularly concerning ontogeny and niche-specific functions, and discuss how these specialized populations become dysregulated in cirrhosis. Additionally, we explore how therapeutic approaches might target these macrophages to restore barrier-protective functions.

## 2 Intestinal barrier defense against bacterial translocation

### 2.1 Intestinal epithelium

The intestinal epithelium constitutes the first line of defense against luminal microbes, forming a highly specialized and dynamic barrier that separates the intestinal lumen from underlying tissue compartments. This defense relies on multiple coordinated mechanisms and specialized epithelial subsets, to reinforce barrier integrity and antimicrobial defense. Importantly, the epithelium actively communicates with underlying immune cells in the lamina propria, particularly macrophages, to balance tolerance toward commensals with rapid responses to pathogens (23, 24).

In homeostasis, epithelial integrity is continuously maintained through rapid turnover of intestinal epithelial cells (IECs) in the crypt base, and is tightly regulated by niche-derived factors, including signals provided by macrophages within the local microenvironment (25).

This highly coordinated renewal process preserves barrier integrity and restricts microbial passage across the intestinal epithelium. Disruption of this barrier increases intestinal permeability, leads to microbial dysbiosis, promotes bacterial translocation, and contributes to chronic inflammation, highlighting the central role of the epithelium in maintaining intestinal homeostasis (23, 24). In cirrhosis, epithelial barrier integrity is further compromised by altered expression of tight junction proteins (e.g., claudins and occludins), resulting in increased permeability and enhanced bacterial passage into underlying tissue compartments (26).

### 2.2 Lamina propria macrophages

The intestinal lamina propria, located beneath the intestinal epithelium, features the most abundant and well-characterized population of macrophages within the gastrointestinal tract (13, 14). Lamina propria macrophages act as essential mediators of host defense and immunity, forming a critical component of the innate immune system. Positioned at the interface between the intestinal tissue and lumen, they continuously respond to dietary and microbial antigens, thereby maintaining a finely tuned balance between pathogen defense, immune homeostasis, and the induction of oral tolerance (13, 27). A key specialized subset within this compartment is formed by subepithelial macrophages, which are strategically positioned directly beneath the intestinal epithelium (Section 2.2.2) (28, 29).

#### 2.2.1 Monocyte-to-macrophage differentiation

Continuously exposed to potentially harmful agents from the intestinal lumen, lamina propria macrophages require constant replenishment from bone marrow-derived monocytes. In mice, Ccr2^+^ Ly6C^+^ monocytes are recruited into the intestinal lamina propria in response to microbial signals (30). Following recruitment, these cells undergo a stepwise differentiation process, passing through phenotypically distinct intermediate stages. This maturation trajectory, progressing from monocytes to highly differentiated tissue macrophages, is commonly referred to as the “monocyte-to-macrophage waterfall” (13, 14). During this process, cells lose Ccr2 and Ly6C expression and acquire expression of major histocompatibility complex class II (MhcII) and Cx3cr1, a chemokine receptor essential for immune surveillance (13, 14, 31). Cx3cr1^+^ macrophages can extend dendritic projections through the epithelial layer to directly sample luminal antigens (32). Ultimately, these cells differentiate into mature macrophages expressing F4/80, Cd64, Cd163, Cd11c, and Cd206 (14, 30, 31).

A comparable differentiation trajectory has been described in humans, where circulating CD14^+^ CD11c^+^ CCR2^+^ monocytes display a progressive decline in CD14, CD11c and CCR2 expression, while acquiring HLA-DR and integrin CD11b expression after tissue entry (33, 34). HLA-DR is a MHCII molecule that mediates antigen presentation to CD4^+^ T cells and is therefore essential for the initiation of adaptive immune responses (35). In both species, microbial components are the principal drivers of the continuous recruitment and maturation process of monocytes (14, 30).

In patients with cirrhosis, however, this differentiation program becomes dysregulated, and macrophages acquire pro-inflammatory features, including increased expression of CD14, iNOS, and TREM1. These macrophages, isolated from the duodenum of cirrhotic patients, exhibit a shift toward a hyperinflammatory state, contributing to chronic inflammation and facilitating bacterial translocation into the lamina propria (Section 2.4) (26).

In addition, reduced HLA-DR expression on circulating monocytes is observed in patients with cirrhosis (36). As HLA-DR is essential for antigen presentation and the initiation of adaptive immune responses, reduced expression may reflect impaired monocyte immune function (35). Consistent with this concept, low HLA-DR expression has been associated with advanced liver disease severity and poor clinical outcomes, particularly in patients with decompensated cirrhosis and ACLF (36–38). However, this evidence is derived from peripheral blood monocytes rather than tissue-resident macrophage populations.

#### 2.2.2 Subepithelial lamina propria macrophages

Among lamina propria macrophages, subepithelial macrophages constitute a specialized population that is strategically positioned directly beneath the intestinal epithelium (13, 28, 29). Subepithelial macrophages act as a first-line immune barrier and, in murine models, have been shown to sample luminal antigens via transcytosis across the epithelium or through transepithelial dendrites - protrusions extended by Cx3cr1^+^ macrophages between epithelial cells in the small intestine (14, 32, 39). In addition, Cd11c^+^ subepithelial macrophages in the murine colon form “balloon-like” protrusions that penetrate the base of the epithelium to monitor absorbed fluids. When these fluids contain fungal metabolites or toxins, the macrophages can inhibit epithelial uptake, thereby preventing apoptosis, limiting inflammation, and preserving intestinal barrier integrity (39).

**Figure 1:**
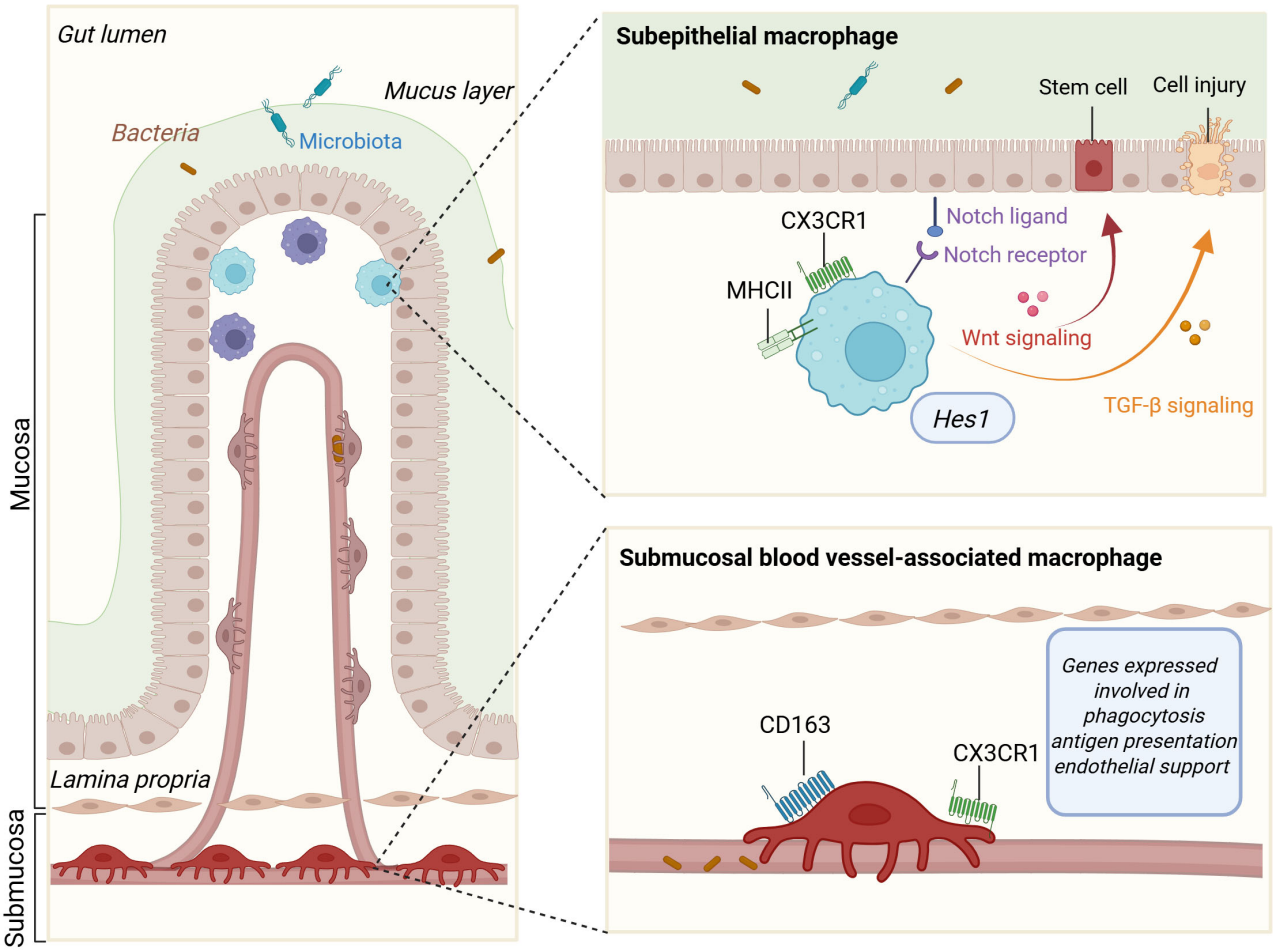
Functional heterogeneity of intestinal macrophages in gut barrier homeostasis. Schematic overview of the intestinal barrier illustrating the spatial distribution and functional specialization of intestinal macrophage subsets. In the mucosa, subepithelial macrophages (CX3CR1^+^, MHCII^+^) are positioned directly beneath the epithelial layer, where they contribute to antigen sampling, immune surveillance, and maintenance of epithelial integrity. A distinct mature subset expressing the Notch target gene *Hes1* (Hes1^+^ macrophages) is enriched within the villus compartment and is associated with pathways involved in epithelial homeostasis and regeneration, including TGF-β, Wnt, and Notch signaling. Through these interactions, Hes1^+^ macrophages contribute to epithelial turnover, repair, and barrier maintenance. In the submucosa, long-lived CD163^+^ perivascular macrophages reside in close proximity to larger blood vessels. They support vascular integrity through phagocytosis, angiogenesis, and maintenance of the gut vascular barrier, thereby limiting the translocation of bacterial components from the lumen into the portal circulation.

Recent single-cell transcriptomic analyses have further delineated the heterogeneity of subepithelial macrophage populations (40). A distinct mature subset characterized by the expression of the Notch-target gene *Hes1* (Hes1^+^ macrophages) has been identified predominantly within the intestinal villi. These Hes1^+^ macrophages display an upregulation of pathways essential for maintaining epithelial homeostasis and turnover, including Transforming Growth Factor-β (TGF-β), Notch, and Wnt signaling, underscoring their critical role in epithelial repair and regeneration (**Figure 1**) (40). TGF-β is expressed by the intestinal epithelium and contributes to epithelial regeneration, while Wnt and Notch are essential for epithelial cell differentiation and spatial organization (23, 25). Importantly, the activity of these pathways is tightly regulated, as excessive or prolonged activation in pathological conditions has been associated with adverse outcomes including fibrosis and tumorigenesis (41–43). Although *Hes1* expression is conserved between mice and humans, its functional characterization remains more extensively defined in murine models, particularly in small intestinal macrophage populations (40).

While Cx3cr1^+^ and Cd11c^+^ subepithelial macrophages are primarily involved in antigen sampling and phagocytosis, Hes1^+^ macrophages are distinguished by their predominant role in regulating epithelial turnover and repair through activation of regenerative pathways (39, 40). Beyond maintaining epithelial homeostasis, this barrier-protective function is critical for preventing bacterial translocation from the intestinal lumen into underlying tissues and the portal circulation. Consequently, dysfunction of Hes1^+^ macrophages is likely to impair epithelial regeneration, facilitate microbial translocation, and contribute to increased susceptibility to infections (Section 2.4) (40).

#### 2.2.3 Additional lamina propria macrophage markers

In murine models, a hallmark of mature lamina propria macrophages is the expression of Timd4, a scavenger receptor involved in the clearance of apoptotic cells (44). Timd4^+^ macrophages are predominantly located near the tips of the villi, where they are in close proximity to the apoptotic cells within the epithelial layer (45, 46). Their strategic positioning allows them to efficiently remove dying cells, thereby contributing to the maintenance of the epithelial barrier. This population also represents a subset of long-lived tissue-resident macrophages within the lamina propria, suggesting a role in sustaining tissue integrity (45).

Another identified population is characterized by Cd121b expression, found to be enriched in the mucosa, around the tips of the villi. These macrophages are also implicated in apoptotic cell clearance and barrier maintenance and are strongly influenced by the microbiota, as their numbers are significantly reduced in germ-free mice (47).

Anti-inflammatory roles of murine lamina propria macrophages are further supported by the expression of Cd206 and Cd163, which are involved in recognition and clearance of microbial and hemoglobin-associated ligands, as well as protection against oxidative stress (48, 49). Cd206 is important for the uptake of mannose-rich glycoconjugates, often derived from commensal bacteria (49). Cd163 is a marker of tissue-resident intestinal macrophages that recognizes Gram^+^ and Gram^-^ bacteria (46).

While direct evidence for alterations of these specific macrophage subsets in cirrhosis is currently limited, their homeostatic functions are highly relevant for understanding barrier integrity in health.

### 2.3 Gut vascular barrier

In addition to the intestinal epithelial barrier that consists of the epithelial monolayer and its associated lamina propria macrophages, the gut is equipped with a gut vascular barrier (GVB, 50). This additional layer of defense is required to protect against invading pathogens translocating into the systemic circulation (13, 50, 51). Under hazardous conditions, harmful enteric pathogens can cross the epithelial barrier and cause GVB damage, which eventually allows the dissemination of bacteria or their constituents into the blood circulation and the liver (52). In mice, different populations of blood vessel-associated macrophages protect the GVB, where each population play a central role in regulating inflammatory responses and preserving vascular integrity (13, 53).

#### 2.3.1 Blood vessel-associated macrophages

In the intestinal villi, a subset of short-lived mucosal perivascular macrophages was described within the villi itself. It was shown that these cells form a complex network around the microcirculation. These macrophages are continuously replenished by circulating Ccr2^+^ cells, and their differentiation depends on the transcription factor Nr4a1 and the presence of the microbiota (13, 53). Importantly, this population is reduced during dysbiosis, resulting in increased systemic dissemination of intestinal pathogens (52, 53). Due to their proximity to luminal contents and blood vessels, these macrophages likely undergo rapid turnover, in contrast to the self-sustaining population in the submucosa (53).

In contrast to the short-lived population in the villi, a distinct group of long-lived, self-maintaining resident macrophages resides in the submucosa (54). These cells are closely positioned to the submucosal vascular plexus, where large veins and arteries branch into the mucosal capillary network. Apart from the bowel-protecting gene *Adamdec1*, these cells express genes involved in angiogenesis, such as *Ecm*, *Tnfaip2*, *Anpep*, *Hif1a*, *Mmp2*, and *Mmp14*. Specific depletion of this self-maintaining, embryonically derived cell population resulted in increased vascular permeability, highlighting their crucial role in maintaining vascular integrity and supporting the GVB (40, 54). Recent spatial transcriptomic studies have confirmed that these long-lived, blood vessel-associated macrophages are characterized by the expression of Cd163 and are enriched for genes involved in receptor-mediated phagocytosis, efferocytosis, and angiogenesis (40, 46).

Additionally, a group of Cd169^+^ macrophages has been identified in close association with the vasculature and lymphatics in the deeper margin of the lamina propria. These bone marrow-derived Cd169^+^ macrophages sense and clear circulating antigens, exhibit strong phagocytic activity, and induce tolerance to antigens from the spleen or lymph nodes (10, 55). They also facilitate monocyte recruitment via the release of C-C motif chemokine ligand 8 (Ccl8) during experimental colitis, and their selective depletion exacerbates mucosal injury, highlighting their role in regulating intestinal inflammation (56).

### 2.4 Intestinal macrophage dysfunction in cirrhosis

Infections in patients with cirrhosis frequently originate from the gastrointestinal tract, where bacteria breach the intestinal barrier and disseminate to the liver and systemic circulation. This barrier dysfunction, combined with impaired immune surveillance, contributes to a chronic hyperinflammatory state that underpins cirrhosis-associated immune dysfunction (10). Multiple factors compromise intestinal barrier integrity in cirrhosis, including small bowel dysmotility, altered bacterial flora, thinning of the mucus layer, reduced secretion of antimicrobial peptides, and altered farnesoid X receptor signaling, a pathway involved in bile acid homeostasis and intestinal immune responses (8–10, 21) However, recent evidence suggests that bacterial translocation is not solely driven by epithelial or luminal changes, but instead reflects failure across several regulatory checkpoints, with a central contribution of macrophage dysfunction within the intestinal immune and vascular niche (26, 40, 54).

Intestinal macrophages are characterized in patients with compensated and decompensated cirrhosis as having an aberrantly activated phenotype (26). Macrophages isolated from the duodenum of cirrhotic patients exhibited increased expression of CD14, iNOS, and TREM1, reflecting a shift toward a hyperinflammatory state. In decompensated cirrhosis, they produced elevated levels of pro-inflammatory cytokines such as IL-6, IL-8, MCP-1, and CCL13, alongside increased nitric oxide production (26). Although these changes reflect heightened immune activation, they do not translate into effective bacterial containment. Instead, persistent macrophage activation contributes to ongoing inflammation and further disrupts the intestinal epithelial barrier, facilitating bacterial translocation into the lamina propria (26).

More recently, bacterial translocation in cirrhosis has been linked to coordinated failure of the intestinal epithelial barrier and the gut vascular barrier, together with profound dysfunction of gut-vascular macrophage populations (40). At the epithelial level, cirrhosis is associated with increased epithelial cell death and focal regions of villus loss, creating potential entry sites for luminal bacteria (40). Hes1^+^ macrophages exhibit marked downregulation of regenerative pathways involved in epithelial repair and homeostasis, resulting in insufficient epithelial regeneration following injury and failure to efficiently restore epithelial continuity. This defective repair response prolongs barrier disruption, thereby increasing the probability of bacterial translocation into the lamina propria and subsequent systemic dissemination (40).

At the vascular level, cirrhosis disrupts macrophage populations that safeguard the GVB, as Cd163^+^ vascular-associated macrophages exhibit impaired bacterial clearance. These macrophages exhibit disrupted interconnectivity around the blood vessels and a loss of submucosal zonation. This loss of zonation and inadequate macrophage coverage along the vasculature may contribute to increased bacterial translocation from the intestinal lumen and tissue into the systemic circulation (40). The precise mechanism underlying this loss of vascular association remains a subject for future investigation, but could potentially involve multiple mechanisms including altered TGF-β signaling, or the lack of macrophage migration inhibitory factor (Mif) or C-C motif chemokine ligand 2 (Ccl2) produced by pericytes (46, 57).

**Figure 2:**
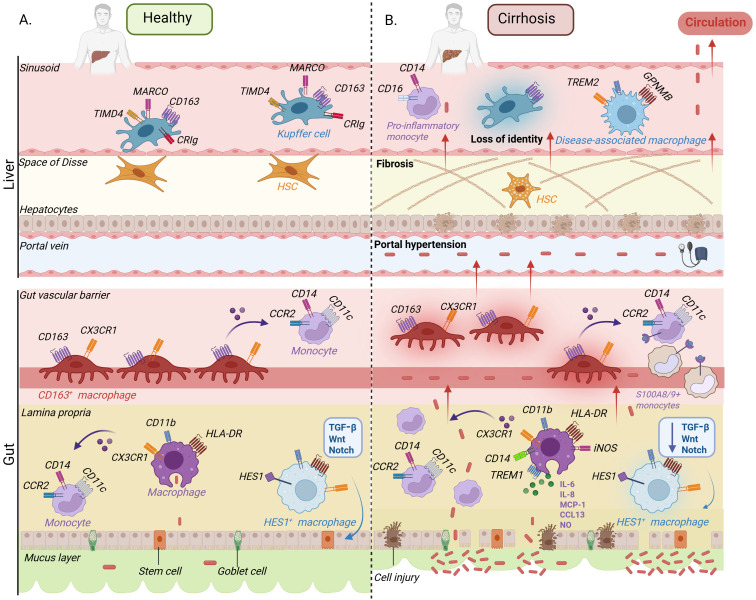
Human macrophage populations along the gut-liver axis in health and cirrhosis. **(A)** Under homeostatic conditions, CCR2^+^CD14^+^CD11c^+^ monocytes continuously replenish intestinal macrophage populations. CX3CR1^+^ and CD11c^+^ macrophages contribute to antigen sampling and phagocytosis, whereas HES1^+^ macrophages support epithelial regeneration through TGF-β, Wnt, and Notch signaling. CD163^+^ vascular-associated macrophages preserve gut vascular barrier function and limit bacterial dissemination. In the liver, Kupffer cells (KCs) characterized by TIMD4, MARCO, CD163, and CRIg expression, form a second immune barrier by efficiently clearing gut-derived pathogens from the portal circulation. **(B)** In cirrhosis, epithelial injury, liver fibrosis, and portal hypertension disrupt the gut-liver axis. Intestinal macrophages acquire a pro-inflammatory phenotype characterized by increased expression of CD14, TREM1, and iNOS, accompanied by accumulation of inflammatory S100A8/9^+^ cells and elevated pro-inflammatory cytokines, including IL-6, IL-8, MCP-1, CCL13, as well as increased nitric oxide (NO) production. HES1^+^ and CD163^+^ macrophages exhibit impaired barrier-supporting functions, facilitating bacterial translocation across the intestinal epithelium and the gut vascular barrier (GVB). In the liver, fibrosis-associated niche disruption leads to loss of KC identity due to impaired interactions with liver sinusoidal endothelial cells (LSECs), hepatocytes and hepatic stellate cells (HSCs), together with accumulation of infiltrating monocyte-derived macrophages and TREM2^+^GPNMB^+^ disease-associated macrophages. Together, these alterations impair bacterial clearance, promote chronic inflammation, and facilitate systemic dissemination of gut-derived bacteria and microbial products, thereby increasing susceptibility to infections, acute decompensation, and acute-on-chronic liver failure.

Importantly, the transcriptional features of Cd163^+^ vascular-associated and Hes1^+^ epithelial-associated dysfunctional intestinal macrophages are conserved between murine models and duodenal biopsies from patients with cirrhosis. In both species, macrophages display dysregulation of pathways involved in vascular remodeling and barrier maintenance, supporting the translational relevance of murine findings (40). In patients with decompensated cirrhosis, this dysfunctional macrophage phenotype is further accompanied by an influx of inflammatory S100A8/9^+^ monocytes and increased IL-1β expression, indicating a shift toward a more pro-inflammatory intestinal immune environment (40). Together, these alterations contribute to barrier failure and promote bacterial translocation into the portal circulation, facilitating the delivery of gut-derived bacteria to the liver (**Figure 2**). It should be noted, however, that the murine analyses encompass broader intestinal regions, whereas the available human data are derived specifically from duodenal biopsies, which may limit direct regional comparisons (40).

## 3 Liver barrier defense against systemic bacterial dissemination

The liver possesses a unique vasculature system in which nutrient-rich blood originating from the intestine enters directly through the portal vein (58, 59). This venous inflow mixes with oxygenated blood from the hepatic artery and passes through an extensive network of sinusoids before draining into the central vein. The hepatic parenchyma is organized into well-defined periportal and centrilobular regions that form repetitive hexagonally arranged lobular units. Specialized parenchymal cells, predominantly hepatocytes, line the sinusoidal endothelium. While this architecture enables efficient nutrient processing, it also poses a risk for systemic infection, as the liver is continuously exposed to gut-derived bacteria and microbial products that must be rapidly cleared to prevent systemic dissemination (58–60).

### 3.1 Kupffer cells as hepatic gatekeepers

Although the liver contains multiple macrophage populations, Kupffer cells (KCs) represent the predominant subset under homeostatic conditions (15, 61). Whether heterogeneity exists within the KC compartment itself remains an ongoing debate. However, given their strong dependence on local microenvironmental cues to maintain their identity, it is highly plausible that KCs exhibit functional and phenotypic diversity across the liver lobule. Classical macrophage markers such as Cd11b, F4/80, or Cd68 are insufficient to unequivocally identify bona fide KCs, particularly during inflammation when monocytes infiltrate the liver and adopt a similar phenotype (15, 16). Instead, a conserved KC gene signature encompassing *Timd4*, *Vsig4*, *Clec4f*, *Cd5l*, *Cd163* along with transcription factors Id3 and Nr1h3, defines their unique identity (61–63). Tim4, in particular, is the most reliable marker for embryo-derived KCs in mice, as it is absent from recently recruited monocyte-derived macrophages (62–64).

KCs are strategically positioned within the lumen of the hepatic sinusoids. This intravascular localization enables them to function as highly efficient filters that clear pathogens, cellular debris, and aged platelets from the circulation within seconds (65, 66). To support this function, KCs express a broad repertoire of receptors involved in pathogen recognition and clearance, including complement receptors, scavenger receptors, Toll-like receptors (TLRs) and other pattern recognition receptors. Pathogen detection is further facilitated by circulating opsonins (e.g., immunoglobulins and complement proteins), which coat microbial particles and enhance their uptake through Fcγ receptor- and complement receptor-mediated mechanisms (61). A central component of this clearance machinery is the complement receptor CRIg, which is highly expressed by KCs and plays a pivotal role in recognizing complement-opsonized targets (61, 67).

**Figure 3:**
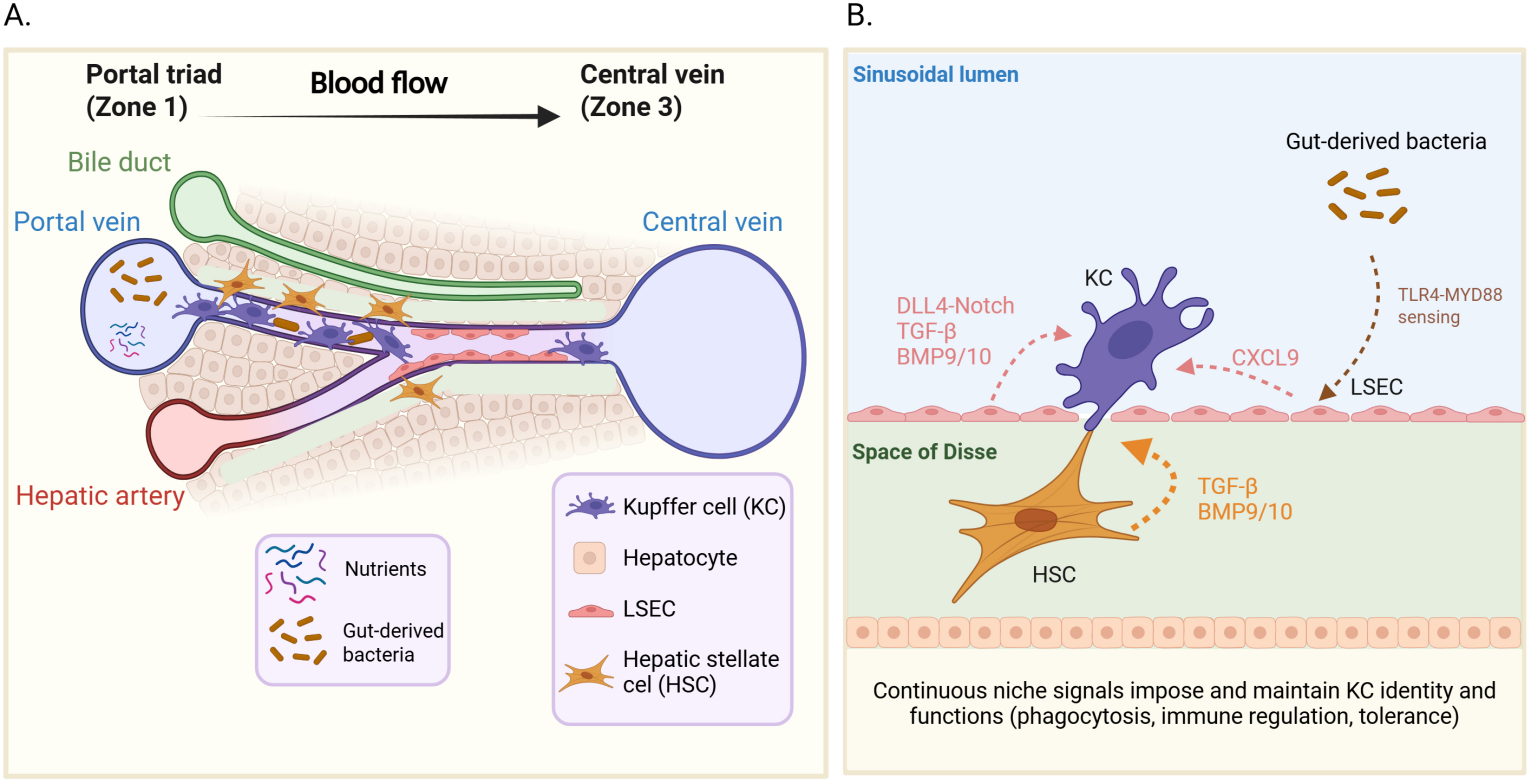
Localization and niche-dependent maintenance of Kupffer cells (KCs) in the healthy liver. **(A)** Schematic representation of the liver barrier and the localization of KCs within the hepatic sinusoids. Nutrient-rich blood from the intestine enters through the portal vein and mixes with oxygenated blood from the hepatic artery in the portal triad (zone 1), before flowing through the sinusoidal network toward the central vein (zone 3). KCs are preferentially enriched in the periportal sinusoidal lumen, where they encounter the highest concentrations of gut-derived bacteria and microbial products arriving via the portal circulation. Their positioning and functional identity are tightly regulated by a specialized hepatic niche composed of liver sinusoidal endothelial cells (LSECs), hepatocytes, and hepatic stellate cells (HSCs), which provide continuous spatial and molecular cues that sustain KC survival, zonation, and immunoregulatory function. **(B)** Close-up of the KC niche within the hepatic sinusoid. KCs extend pseudopods through fenestrated LSECs into the space of Disse, maintaining close contact with HSCs and hepatocytes. KC identity and function are sustained by continuous niche-derived signals, including DLL4-Notch signaling from LSECs, TGF-β, and BMP9/10 from LSECs and HSCs, as well as CXCL9-mediated periportal positioning induced by commensal-derived TLR4-MyD88 signaling.

Despite their intravascular localization, KCs extend their pseudopods through the fenestrations of liver sinusoidal endothelial cells (LSECs) into the perisinusoidal space of Disse, ensuring continuous contact with parenchymal cells (63, 68). This high level of interaction with LSECs, hepatocytes, and hepatic stellate cells (HSCs) constitutes the KC niche (61–63). The niche provides critical morphogenic signals - including DLL4-Notch signaling from LSECs, TGF-β, and BMP9/10 from HSCs and LSECs - that collectively impose and maintain KC identity, function and spatial organization (**Figure 3**) (62, 63).

Due to the decreasing gradients of gut-derived endotoxins, oxygen, and nutrients along the sinusoids, KCs exhibit functional and phenotypic zonation (16, 61, 62). In mice, KCs are preferentially localized in the periportal regions (zone 1), where they encounter the highest concentration of gut-derived substances (**Figure 3A**) (62, 69). This spatial positioning is driven by a chemokine C-X-C motif ligand 9 (CXCL9) gradient retained in the endothelial glycocalyx, which is dependent on commensal-derived endotoxin sensing via TLR4-MyD88 signaling by LSECs (**Figure 3B**) (69). KCs in the periportal zone are larger and exhibit significantly higher phagocytic activity compared to those in pericentral areas, serving as the frontline defense against translocating bacteria (59, 69). A distinct subset of KCs expressing the scavenger receptor Marco and anti-inflammatory genes (e.g., *IL-10*) is concentrated in these periportal zones and demonstrates preferential bacterial uptake (69). Similarly, single-cell transcriptomic analyses of the human liver have identified a conserved macrophage subset expressing MARCO in periportal areas, aligning with the functional zonation observed in mice (62).

### 3.2 Hepatic macrophage dysfunction in cirrhosis

#### 3.2.1 Loss of Kupffer cell identity and spatial niche disruption

Cirrhosis is characterized by profound structural changes in the liver, including hepatocyte loss, extensive fibrosis, and collagen deposition around the sinusoids (70, 71). These architectural alterations lead to the redistribution of blood flow away from the sinusoids through the formation of portosystemic shunt vessels, substantially reducing the exposure of blood-borne pathogens to KCs and thereby impairing their clearance capacity (**Figure 2**) (71–73). Furthermore, this remodeling disrupts the critical cellular interactions within the KC niche required to maintain their functional identity (73).

Recent advances in single-cell RNA sequencing (scRNA-seq) and spatial transcriptomics in murine models have provided unprecedented resolution into the fate of KCs during fibrogenesis. In the healthy liver, KCs are primarily zonated within the periportal (zone 1) regions of the sinusoids, where they act as the primary firewall against gut-derived pathogens (69). Spatial proteogenomics has revealed that this positioning is maintained by a specialized niche of hepatocytes, endothelial cells, and hepatic stellate cells that imprint KC identity (62). However, during the development of liver fibrosis, KCs exhibit altered morphology and a marked reduction in pseudopods, contributing to the disruption of these essential niche interactions. Consequently, KCs lose key maturity markers, such as Clec4f, Tim4, and CRIg, accompanied by significant functional impairment (73).

This loss of KC identity has been observed in liver biopsies from patients with cirrhosis (72, 73). Crucially, while scRNA-seq studies consistently report a profound depletion of KCs in advanced cirrhosis, recent comparative transcriptomic analyses caution that this may be partially exacerbated by technical artifacts (74, 75). For instance, Van Melkebeke et al. demonstrated that scRNA-seq protocols disproportionately fail to recover fragile KCs from transjugular liver biopsies of decompensated patients due to dissociation-induced stress, whereas single-nucleus RNA sequencing (snRNA-seq) provides a more accurate representation of the surviving myeloid landscape (75). Nonetheless, integrating snRNA-seq with spatial transcriptomics confirms a genuine and significant loss of resident KCs and their periportal zonation as the fibrotic niche expands (73, 75, 76).

#### 3.2.2 Emergence of disease-associated macrophage subpopulations

Beyond the quantitative loss and functional impairment of embryo-derived KCs, chronic liver disease induces profound immunological reprogramming and the emergence of distinct, disease-associated macrophage subpopulations (8, 72). High-resolution scRNA-seq of the human fibrotic niche originally identified the emergence of scar-associated macrophages (SAMs) that expand significantly during cirrhosis. These SAMs, which differentiate from circulating monocytes, are characterized by the expression of TREM2 and CD9, and are topographically restricted to the fibrotic scar (74).

However, the macrophage landscape is even more complex and dynamically regulated by spatial and metabolic contexts. A recent study by our group utilizing snRNA-seq, spatial multi-omics (GeoMx and CosMx), and iterative protein multiplexing (MILAN) in human biopsies across the MASLD spectrum revealed a massive shift in macrophage composition (77). As disease progresses from simple steatosis to active steatohepatitis (MASH) and fibrosis, the classical KC pool (identified by MARCO, CD5L, and TIMD4) is progressively lost and replaced by highly phagocytic, metabolically active macrophages (MetMacs, 77).

These MetMacs are prominently characterized by the expression of GPNMB and TREM2, alongside lipid and cholesterol metabolic markers such as HS3ST2, LPL, and FABP5. Spatial transcriptomics and multiplexed proteomics demonstrate that these GPNMB^+^ macrophages accumulate specifically in areas of advanced fibrosis, lipogranuloma formation, and cellular debris, where they mediate the clearance of lipid droplets and dying cells. This adaptive metabolic phenotype is tightly regulated by the local spatial context, with GPNMB^+^ MetMacs often supported by IL-32-producing hepatocytes in the inflammatory niche (77).

The transition to MASH and advanced fibrosis is thus marked by the decline of homeostatic KCs and the parallel expansion of these GPNMB^+^ TREM2^+^ MetMacs, which share significant transcriptional overlap with previously described lipid-associated macrophages (LAMs) and SAMs (74, 77).Notably, the abundance of these GPNMB^+^ macrophages correlates strongly with disease severity and lobular inflammation, suggesting they are critical drivers of the adaptive immune response during fibrogenesis.

#### 3.2.3 Compensatory mechanisms: monocyte recruitment and syncytia formation

When the hepatic immune surveillance is compromised and embryo-derived KCs undergo cell death or loss of identity, there is a compensatory recruitment of circulating monocytes, particularly the pro-inflammatory CD14^+^ CD16^+^ subset, to the liver (38, 78, 79). These recruited monocytes differentiate into monocyte-derived macrophages that attempt to replace the KC pool and are characterized by increased phagocytosis, antigen presentation and secretion of pro-inflammatory cytokines, including TNF-α, IL-6, IL-8, and IL-1β (78, 79).In fibrotic livers, where sinusoids are rarefied and circulation is redirected through high-flow collateral vessels, these recruited cells adapt by forming KC-like syncytia - large multi-nucleated clusters located in abnormally large vessels - that partially restore filtration functions and clear bloodborne bacteria via CRIg expression. The formation of these syncytia depends on bacterial signals leaking through a permeable gut barrier, as well as the cell adhesion molecule CD44 and the scavenger receptor CD36 (73).

However, these compensatory responses remain incomplete in cirrhosis. Monocyte-derived macrophages fail to fully recapitulate the spatial positioning, phagocytic efficiency, and regulatory functions of resident KCs, as they require significant time and intact niche signals to fully mature (73). Furthermore, circulating monocytes in cirrhosis exhibit impaired Fcγ receptor function, which is critical for the clearance of IgG-coated bacteria, further exacerbating the susceptibility to infection (80).

Additionally, recent spatial transcriptomic profiling has revealed that the TREM2^+^ LAM phenotype is not exclusively restricted to recruited monocytes. De Ponti et al. demonstrated that a subset of resident KCs can also adopt a LAM-like state to assist in efferocytosis and the resolution of fibrosis during disease regression (64). While these adaptive macrophage states (including GPNMB^+^ MetMacs, SAMs, and LAMs) play a critical role in tissue repair and lipid clearance, their massive accumulation at the expense of classical KCs ultimately leaves the cirrhotic liver vulnerable to systemic bacterial dissemination.

## 4 Targeting macrophage responses

### 4.1 Current therapeutic approaches targeting macrophage dysfunction

Current strategies to prevent bacterial infections in cirrhosis rely primarily on antibiotic prophylaxis, which fails to address the underlying immune dysfunction and is associated with high mortality and the emergence of antimicrobial resistance (20, 21). Despite the central role of macrophages in barrier defense and antimicrobial immunity, direct therapeutic targeting of these cells in cirrhosis remains a major challenge. The clinical translation of such strategies is hindered by the need to selectively modulate macrophage function without inducing generalized immunosuppression, which would increase infection risk, or excessive immune activation, which could exacerbate systemic inflammation and organ injury (8). Consequently, treating the immune dysfunction in cirrhosis and ACLF demands precise, context-dependent immune modulation.

In ACLF, macrophage dysfunction may represent a potentially reversible component of immune paralysis. Patients with ACLF exhibit increased numbers of immunoregulatory monocytes and macrophages expressing the receptor tyrosine kinase MERTK in the circulation, liver, and lymph nodes (8, 81). MERTK plays a key anti-inflammatory role by promoting the clearance of apoptotic cells and downregulating the innate immune response to microbes (82). Elevated MERTK expression correlates with disease severity, impaired antimicrobial immune responses, and a higher risk of secondary infections (81). Notably, *ex vivo* inhibition of the MERTK signaling pathway using the small-molecule MERTK inhibitor UNC569 restored monocyte responsiveness to bacterial components, suggesting that modulation of the MERTK pathway may represent a promising therapeutic strategy in ACLF (8, 81). However, evidence remains limited and is largely derived from preclinical and *ex vivo* studies, warranting further investigation before clinical translation.

Beyond pharmacological modulation, cellular therapies are emerging as a promising frontier. A recent Phase 2 open-label randomized controlled trial investigated the use of autologous monocyte-derived macrophage therapy in patients with compensated liver cirrhosis (17). In this approach, bone marrow–derived monocytes are isolated from the patient (n=24 control, n=27 therapy), differentiated and matured *ex vivo* into mature macrophages, characterized by high CD14, 25F9 expression, together with sustained expression of markers associated with tissue repair and inflammation resolution, including CD206, CD163, and CD169. The study demonstrated that the infusion of *ex vivo* matured autologous macrophages is safe and well-tolerated over a 12-month follow-up period. Furthermore, the therapy showed potential efficacy in reducing liver fibrosis as assessed by vibration-controlled transient elastography (VCTE), alongside improvements in liver disease severity as reflected by MELD score, suggesting that replenishing the liver with functional macrophages could counteract the progressive loss of resident KC function (17).

Importantly, long-term follow-up data have recently extended these findings, reporting a sustained reduction in the risk of death or liver transplantation over a 4-year period compared with standard medical care, providing additional support for macrophage-targeted therapeutic approaches in chronic liver disease (19).

### 4.2 Future therapeutic opportunities within the gut-liver axis

In the context of the intestinal barrier, the recent identification of specific gut-resident macrophage subsets opens novel, highly targeted therapeutic avenues. Specifically, the epithelial-associated Hes1^+^ macrophages, which orchestrate epithelial repair via TGF-β, Notch, and Wnt signaling, represent a highly promising target. While currently speculative, therapeutically restoring the function of these Hes1^+^ macrophages could fundamentally alter the management of cirrhosis-associated infections. By pharmacologically agonizing the Notch or Wnt pathways locally within the gut mucosa, or by utilizing targeted nanoparticle delivery systems to deliver pro-regenerative cytokines (such as TGF-β or IL-22) directly to these cells, it may be possible to reinvigorate their capacity for epithelial regeneration. Such an approach would directly counteract the aberrant epithelial cell death and focal villus loss that serve as primary entry points for bacterial translocation. Unlike broad-spectrum antibiotics or systemic immunosuppressants, restoring Hes1^+^ macrophage function would fortify the physical intestinal barrier from within, preventing bacterial ingress at its source while preserving systemic immune competence. However, given the pleiotropic and context-dependent functions of TGF-β, Wnt, and Notch signaling, therapeutic modulation of these pathways would require careful control, as excessive or prolonged activation may promote fibrosis, aberrant tissue remodeling, or tumorigenesis (41–43).

Collectively, while current evidence supporting macrophage-targeted therapies in cirrhosis-associated infections is still evolving, these findings suggest that macrophages represent a modifiable component of cirrhosis-associated immune dysfunction. Addressing the critical knowledge gaps regarding macrophage heterogeneity, plasticity, and niche-specific requirements will be essential for designing interventions that can safely restore immune barrier function and reduce infection-related complications in advanced liver disease.

## 5 Conclusion

Cirrhosis is characterized by a profound disruption of the gut-liver axis, resulting in increased bacterial translocation, chronic inflammation, and a high susceptibility to infections that drive acute decompensation and ACLF. Throughout this review, we have highlighted the central role of intestinal and hepatic macrophages as specialized cellular gatekeepers of the epithelial, vascular, and hepatic immune barriers. Under physiological conditions, these macrophage populations are ontogenetically distinct, spatially organized, and highly adapted to their specific niches to balance immune tolerance with rapid antimicrobial defense.

In cirrhosis, this sophisticated system becomes progressively dysregulated. Intestinal macrophage function is impaired, with defective epithelial support limiting regeneration and compromised vascular-associated macrophages weakening the gut-vascular barrier. In parallel, KCs lose their spatial organization, phagocytic capacity, and unique identity due to severe architectural distortion and the disruption of niche-derived signals. Although compensatory recruitment of monocyte-derived macrophages occurs, these cells are functionally immature and fail to fully restore effective immune surveillance, culminating in cirrhosis-associated immune dysfunction and increasing infection risk.

Together, these findings position macrophage plasticity and niche dependency as central determinants of barrier failure in cirrhosis. Therapeutic strategies aimed at restoring macrophage function along the gut-liver axis therefore represent a promising approach to strengthen barrier integrity and reduce infection-related complications in advanced liver disease.
